# Autonomic Recalibration: A Promising Approach for Alleviating Myofascial Pain Explored in a Retrospective Case Series

**DOI:** 10.7759/cureus.52450

**Published:** 2024-01-17

**Authors:** Bob Seton, Rishika Pandey, Mary K Piscura, William G Pearson

**Affiliations:** 1 Department of Biomedical Research and Affairs, Edward Via College of Osteopathic Medicine, Auburn, USA

**Keywords:** pain neutralization technique, myofascial trigger point, primal reflex release technique, neuroplastic adaptations, hypervigilance, musculoskeletal system, manual therapy methods, non-opiate pain control, dysfunctional pain, autonomic recalibration

## Abstract

This retrospective case series introduces autonomic recalibration (AR) as a novel approach for alleviating chronic myofascial pain. The manuscript explores the rationale, theory, and practice of AR, which targets the autonomic nervous system (ANS) to restore homeostasis and reduce pain. The involvement of the ANS in pain modulation and the role of autonomic imbalance in chronic pain are discussed, emphasizing the potential benefits of addressing autonomic dysregulation through AR. The technique combines manual interventions and patient education, relying on neuroplastic adaptations. Three diverse case reports are presented to illustrate the effectiveness of AR in patients with different sources of pain. Each case presents a unique clinical scenario, including a nine-year-old male diagnosed with spondylolisthesis, a 68-year-old male with a history of abdominal surgeries, and a 56-year-old male with chronic low back pain following lumbar fusion surgery. In all cases, AR resulted in pain relief, improved sleep, and restoration of functional abilities. These findings support the potential of AR as an effective alternative approach for myofascial pain. Further research is warranted to validate these outcomes and investigate the underlying mechanisms of AR.

## Introduction

The involvement of the autonomic nervous system (ANS) in acute and chronic pain is well documented. Acute pain activates sympathetic arousal, which acts as a stress-induced analgesic and alleviates pain. This pain suppression is mediated by the activation of descending antinociceptive pathways [1]. Conversely, sustained sympathetic activity can result in chronic pain [2]. A prolonged adaptive sympathetic response leads to inadequate muscle relaxation and blood flow associated with chronic pain [3]. On the other hand, decreased parasympathetic nervous system (PNS) activity as indicated by reduced heart rate variability has been associated with increased chronic pain [4]. Autonomic imbalance (increased sympathetic nervous system (SNS) and decreased PNS activity) is known to be associated with a higher degree of pain in patients with conditions such as diabetic peripheral neuropathy and fibromyalgia when compared to patients with the same condition that present with less or no pain symptoms [5-7]. This connection between the ANS and nociceptive pathways makes sense when considering the high level of functional network connectivity in the central nervous system between the autonomic, limbic, and nociceptive processing pathways in the brain [8].

Several manual techniques have been developed to address chronic musculoskeletal pain. Myofascial trigger points are hypersensitive spots in taut bands of skeletal muscle. These trigger points are thought to be affected by autonomic imbalance displaying exacerbated local and referred pain due to sympathetic hyperactivity [9]. Morikawa et al. found that compression at myofascial trigger points significantly increased parasympathetic activity while decreasing sympathetic activity and subjective pain [10]. Using balanced ligamentous, balanced membranous, and cranio-sacral techniques, Ruffini et al. showed that osteopathic manipulative treatment (OMT) can increase parasympathetic function and decrease sympathetic activity in healthy, asymptomatic individuals [11]. Physiotherapy sessions using joint mobilization, soft tissue techniques, a stretching program, and motor control exercises resulted in an increase in PNS activation and a decrease in subjective pain perception in patients suffering from chronic low back pain [12]. Additionally, the use of myofascial release techniques common among manual practitioners releases soft tissue from muscle spasms by stimulating mechanoreceptors in the fascia. It is believed that these mechanoreceptors stimulate the ANS, specifically with a decrease in sympathetic tone, which contributes to a restoration of connective tissue homeostasis and subsequent relaxation of muscle fibers [13]. These manual treatments find common ground in addressing chronic pain by targeting myofascial tissues and their impact on the ANS. A different approach to chronic pain would be the inverse: targeting the ANS to assess its impact on myofascial tissues.

Autonomic recalibration (AR) is a treatment paradigm that targets the ANS to reduce sympathetic dominance and alleviate chronic myofascial pain. Patterns of sympathetic dominance are identified through a head-to-toe palpation exam. The AR approach utilizes multiple manual techniques to stimulate afferent feedback loops in the peripheral nervous system to inhibit sympathetic dominance and restore homeostasis. A trauma history is obtained, and the emotional effect is noted. Where emotional tags felt to contribute to chronic pain are identified, patient education and behavioral treatment are used to address traumas as the source of autonomic dysregulation. The aim of the present study is to describe the theory and practice of AR and provide three retrospective case reports of how patients with varied sources of pain responded to treatment.

Theory

The context of pain, influenced by previous experiences, affects the activation of the SNS, resulting in either the suppression or amplification of pain [1]. Increased sympathetic tone and decreased parasympathetic activity at rest, in addition to increased stress and fatigue, have been reported in patients with chronic musculoskeletal pain [5]. Chronic myofascial pain may then be understood as a dysfunction of ANS information processing, a condition referred to as “dysautonomia”.

Restoring ANS homeostasis mitigates myofascial pain but may also improve gastrointestinal function, immune response, and sleep function behaviors [14]. We theorize that neurologically retained threats create sympathetic hypervigilance throughout the body that can be identified and reduced by the manipulation of reflex loops to eliminate dysfunctional hypervigilance and, subsequently, dysfunctional pain. Furthermore, pain alleviation is sustained through neuroplastic adaptations with the exception of when emotional drivers of pain are identified. When an individual has unresolved emotions from past experiences, such as fear-dominant behaviors from chronic abuse, resetting hypervigilant protective primal reflexes is not enough to allow for complete resolution of pain symptoms. Additional interventions to help process the emotionally learned protective mechanisms associated with the past traumas are subsequently necessary. In summary, the AR approach employs multiple manual and self-regulated techniques to address pain associated with post-traumatic learned autonomic hypervigilance.

## Case presentation

Technique

**Figure 1:**
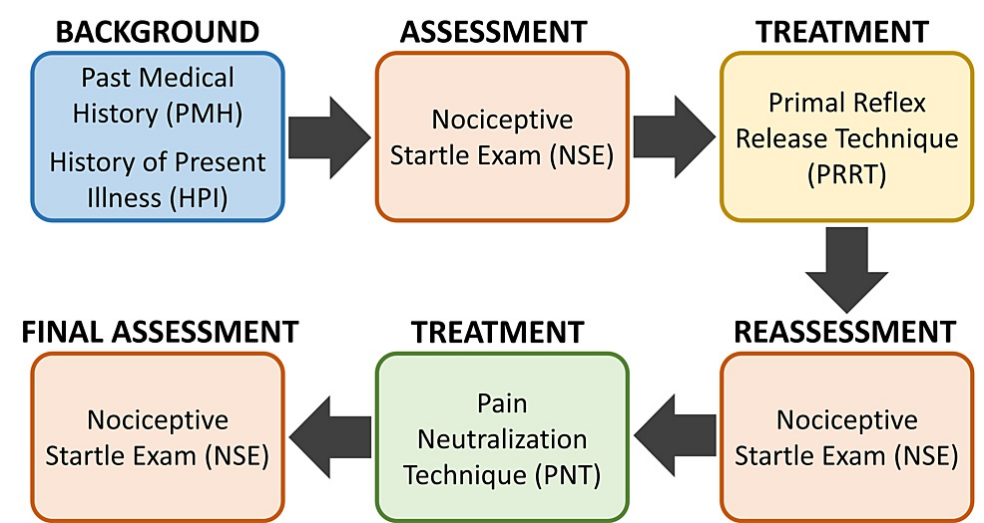
The AR Paradigm Treatment progression for each patient visit includes the background of the patient, assessment of palpable pain through the nociceptive startle exam (NSE), and first treatment using the primal reflex release technique (PRRT). The patient is then reassessed for persistent palpable pain that is treated using the pain neutralization technique (PNT). Success of the treatment is confirmed in a final assessment of NSE. The figure was conceptualized and created by authors BS and MKP, respectively. AR: Autonomic recalibration

The AR approach framework includes the patient medical background, assessment of areas of pain, and subsequent treatment of these areas (Figure 1). The initial visit begins with a history and focal physical exam based on the patient's primary complaint. The patient is evaluated for other complaints that might indicate autonomic dysfunction, including gastrointestinal pathology, autoimmune pathology, and dysfunctional sleeping behaviors. Additionally, there is always an initial orthopedic examination to rule out evidence of structural dysfunction or somatic dysfunction consistent with cancer, tumors, neurological disorders, or recent orthopedic trauma. Patients are asked to describe their pain intensity using a validated and widely used pain questionnaire (PQ), called the numeric rating scale. This PQ has the patient verbally define their pain from 0 to 10, with 0 being “no pain at all” to 10 being “worst pain imaginable".

**Figure 2:**
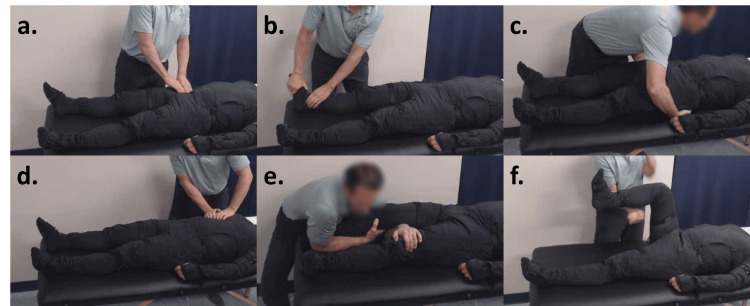
Examples of NSE and PRRT The treating therapist assesses for palpatory hypervigilance during NSE of the right iliopsoas muscle (a), right tarsal tunnel (b), and bilateral piriformis (c). Using PRRT, the treating therapist can be seen applying pressure in various techniques to release the right diaphragm (d), right sacroiliac joint (e), and right hamstring muscles (f). The figure was conceptualized and created by authors BS and MKP, respectively. NSE: Nociceptive startle exam; PRRT: primal reflex release technique

Once the orthopedic exam is completed, a pattern of sympathetic dominance throughout the musculoskeletal system is identified for treatment through a head-to-toe palpation assessment, called a nociceptive startle exam (NSE). The NSE is performed by probing for any atypical reactions to normal touch in which the body withdraws or moves defensively, such as a sudden flex or jerking motion. NSEs are conducted using areas of hypervigilance based on an understanding of dominant primal reflexes as determined by John Iams, PT, and are utilized as the primary assessment tool for his treatments. Examples of the NSE are included in Figures 2a-2c. The first line of treatment utilizes the primal reflex release technique (PRRT), also developed by John Iams. The PRRT is a global autonomic reset to inhibit upregulated primal reflexes, focusing on flexor withdrawal and startle response, which may underlie chronic pain [15,16]. This treatment includes several techniques to reset reflexes and release tension including manual pressure, joint compression, positional release, and tapping targeted reflex loops. Following these manual techniques, in the context of an AR approach, patients recall various traumas over their life span. As they recall these traumas, they employ a physical breathing technique intended to counter a startle response. This combination of physical and mental recall of traumatic experiences is designed to reciprocally inhibit the hyperreactive reflexes and induce relaxation through a reset of the nervous system. Following this global reset of the ANS, sympathetic dominance is then reassessed, and residual nociceptive triggers are noted. Examples of the PRRT being performed can be seen in Figures 2d-2f.

Residual nociceptive trigger points are then treated using the pain neutralization technique (PNT) to eliminate areas of hypervigilance stored in musculoskeletal tissue [17]. The PNT is an alternative therapy approach developed by Dr. Stephen Kaufman designed to reduce neurologically driven reflex patterns presumed to underlie myofascial imbalances, restricted movement, and pain. While stimulating and focusing patients on pain points, the practitioner applies gentle manual pressure to specified soft tissue points. These points are designed to stimulate the Golgi tendon organs and muscle spindles to normalize reflex patterns that contribute to hypertonicity and associated pain. This is repeated until the patient is consciously aware of pain elimination, meaning all areas of hypervigilance, which can be determined by persistent startle reflexes in palpatory examination, have been resolved. Following the initial visit, patients are asked to maintain proper hydration and to refrain from any strenuous physical activity or any activities that could over-excite the nervous system. Sleep, important to facilitate neuroplastic changes encoding for restored homeostasis, is encouraged [18].

A follow-up visit, preferably the subsequent day, begins with a history to determine changes in pain complaints, stomach function, sleep performance, and any other awareness of positive changes. An orthopedic examination is conducted to determine the remaining restriction or pain associated with movement. A head-to-toe NSE is conducted, and any reemerging nociceptive or hypervigilant myofascial tissues are identified and reduced through manual stimulation of reflex circuits. This visit also includes a more progressive and aggressive palpatory inspection of any areas remaining hypervigilant. Areas of scar tissue formation from past injuries and surgeries, as well as individual ligament anatomy surrounding previous injuries or areas of noticeable pain, are all examined more thoroughly compared to the initial visit.

Persistent refractory pain following the reduction of nociceptive trigger points and restoration of a full range of motion may be an indicator of an emotional driver of ANS dysregulation [19]. A patient’s subjective emotional interpretation of pain alleviation following treatment is noted. Close attention is paid to an individual’s facial expressions, choice of verbiage to describe his or her experience, voice tone, and inflection to evaluate affect. As part of each visit, clients are educated about the ANS. This discussion focuses on how physical and emotional trauma can be stored as procedural memories and remain unprocessed based on the emotional tag at the time of the trauma [20]. Autonomic interpretation of external stimuli may trigger unprocessed traumatic memory and elicit a protective mechanism contributing to sympathetic dominance and associated pathology. Based on the patient’s trauma history and personality traits, the patient is instructed to begin certain home treatment techniques. These may include neurologic deep breathing, eye movement desensitization and reprocessing therapy, or other techniques. Patients are educated on their capacity to self-regulate and promote neuroplastic responses by taking voluntary control of this involuntary network. For example, patients are informed that their pain may or may not be a response to structural or biomechanical damage, but rather could be a dysfunction in the processing of information within their "fight or flight" system. This dialogue informs the patient that they can have subconscious inputs that may trigger a chronic pain state, which is reversible.

Case #1

A nine-year-old male presented at a physical therapy (PT) clinic with complaints of back, hip, and knee pain. The patient's history revealed two previous episodes of low back pain after playing at trampoline parks. A recent basketball game exacerbated his symptoms, leading to the diagnosis of spondylolisthesis and spina bifida occulta. The orthopedic surgeon recommended wearing a brace during physical activity and referred the patient for PT. No other medical history was reported. The patient described his pain as constant but variable in intensity, exacerbated by physical activity. Rest, over-the-counter anti-inflammatory medications, heat, and a back brace provided some relief, but the patient had discontinued sports activities for the past 30 days due to pain.

**Figure 3:**
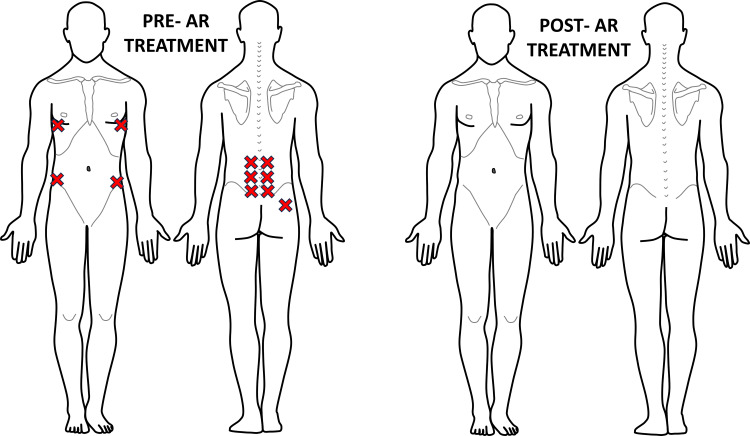
Recording of Case #1 areas of palpatory pain pre- and post-first AR treatment Eleven areas of palpatory pain were recognized pre-AR treatment. Post-AR treatment, this patient displayed 0 areas of hypervigilance, showing complete resolution of this patient's pain concerns. The figure was conceptualized and created by authors BS and MKP, respectively. AR: Autonomic recalibration

PT orthopedic evaluation demonstrated a normal gait without guarding and no pelvic obliquity. Lumbar active range of motion was limited in all planes, with increased pain during lateral rotation and extension of the lumbar spine. Hip range of motion was within normal limits for flexion and abduction, while straight leg raise was restricted bilaterally to 50°. The AR treatment protocol was then implemented. An AR assessment identified 11 areas of hypervigilance, including the bilateral multifidus, bilateral quadratus lumborum, bilateral diaphragm, bilateral serratus anterior, bilateral abdominal obliques, and right gluteus maximus. The PRRT was performed, followed by an NSE reassessment through a palpatory examination. The PNT was applied to eliminate any remaining areas of palpable pain. Subsequently, all areas of reactivity resolved, and the patient reported no pain as indicated by a PQ of 0/10 and full lumbar mobility was restored. During the second visit one week later, the patient again confirmed the absence of pain with PQ of 0/10 and was able to return to playing baseball without discomfort. AR reassessment of sympathetic dominance revealed an 80% improvement in areas of hypervigilance. The bilateral diaphragmatic tenderness that remained was successfully treated using PRRT and PNT techniques (Figure 3). The patient was instructed to resume full sports activity and follow up if any pain symptoms recurred.

One year later, the patient returned with an insidious onset of bilateral hand, foot, calf, and tibial pain. Blood tests indicated rheumatoid arthritis, and subsequent evaluation at an academic hospital led to a diagnosis of amplified musculoskeletal pain syndrome, with concerns of potential wheelchair dependency. An initial assessment revealed the patient reporting a PQ of 5/10 at worst, with symptoms being worse in the morning. The patient had discontinued all sports activities. Recent traumas, including two falls resulting in a facial injury and a concussion, were reported by the parents.

Despite the presenting pain, the patient exhibited full and painless lumbar mobility in all directions, with 5/5 strength in all lower extremity muscle groups. Functional assessments, including squatting and performing lunges without pain, demonstrated unrestricted functionality. ANS examination revealed thirteen areas of hypervigilance, primarily along the diaphragm. The AR treatment plan included PRRT, NSE reassessment, and PNT application for any residual nociceptive points. Additionally, prosody, or vocal patterns that may convey feeling, and emotional disposition were considered during treatment. All areas of hypervigilance resolved after treatment, resulting in a PQ of 0/10. The patient was advised to rest for 24 hours, followed by a resumption of sports activities, with a follow-up scheduled after two days.

**Figure 4:**
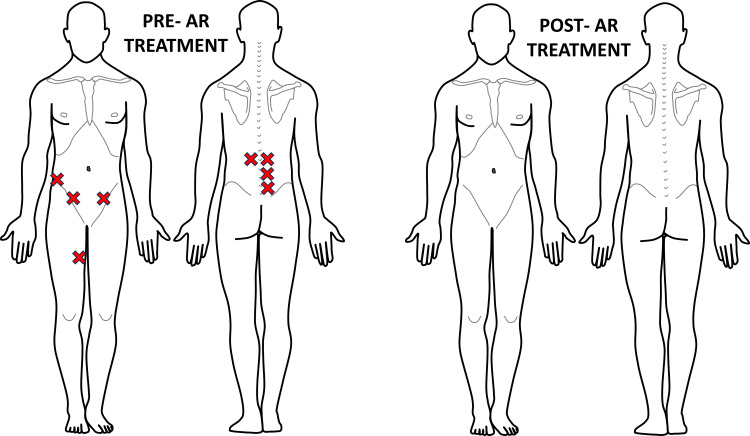
Recording of Case #1 areas of palpatory pain pre- and post-second AR treatment Eight areas of palpatory pain were recognized pre-AR treatment, a reduction from the previous visit. Post-AR treatment, this patient once again displayed 0 areas of hypervigilance. The figure was conceptualized and created by authors BS and MKP, respectively. AR: Autonomic recalibration

During the second visit, the patient reported mild bilateral hamstring achiness when running on the beach, but he was able to resume football practice without pain. A reduced number of areas of hypervigilance (eight) were observed during the assessment. Treatment with PRRT and PNT successfully resolved all areas of hypervigilance (Figure 4). Following treatment, the patient did not experience muscle pain during any functional activities, including hopping and jumping. Instructions were provided to resume full sports activity, with subsequent follow-up as needed. Over the course of the next three years, the patient received five visits for treatment following specific sporting accidents, and each time, symptoms were resolved within a single treatment session.

Case #2

A 68-year-old male presented with chronic right lower abdominal pain that had persisted since a burst appendix in 2009. Subsequent surgeries, including infection clearance, hernia repairs, enlarged pancreatic duct surgery, gallbladder removal, and scar tissue removal, were performed due to complications and persistent pain. Despite taking timed-release morphine daily, the patient described his pain as constant and rated it 7/10 at worst and 3/10 at best. He reported difficulty falling asleep, acid reflux, and reliance on morphine for the past four years.

Physical examination revealed pain during cervical extension, flexion, and right rotation. The cervical range of motion was significantly restricted, and left shoulder pain was experienced at the end ranges of flexion and abduction. Trunk and hip range of motion were within normal limits and pain-free. NSE identified seven areas of hypervigilance, with the right psoas muscle exhibiting the highest reactivity. All areas of hypervigilance were tender, with palpation of the diaphragm bilaterally eliciting a withdrawal response. The PRRT and PNT were applied to address areas of hypervigilance.

**Figure 5:**
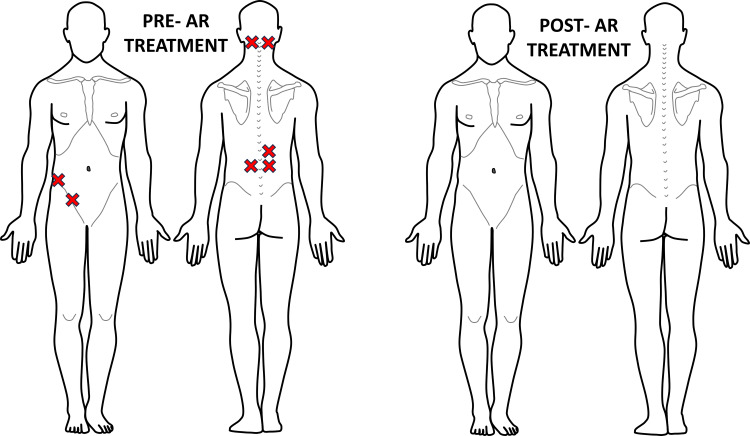
Recording of Case #2 areas of palpatory pain pre- and post-first AR treatment In this patient, seven areas of palpatory pain were recognized pre-AR treatment during their first visit. Post-AR treatment, there were 0 palpable areas of hypervigilance noted. The figure was conceptualized and created by authors BS and MKP, respectively. AR: Autonomic recalibration

After the initial treatment session, the patient reported complete restoration of cervical range of motion to within normal limits and pain-free in all planes. Left shoulder mobility was also restored within normal limits and pain-free. Tenderness in all areas of hypervigilance resolved and PQ was 0/10 (Figure 5). The patient was sent home with instructions to rest and drink water while anticipating improved sleep quality and reduced reliance on morphine.

During the second visit, 24 hours later, the patient reported significant improvement, discontinuing morphine use, and experiencing a good night's sleep. Objective reassessment confirmed maintained cervical range of motion and pain-free motion. However, the lumbar extension range of motion elicited a pulling sensation in the right anterior hip. NSE assessment revealed the persistence of hypervigilance and withdrawal response in the bilateral diaphragm area and tenderness in the right psoas. Although the patient reported less subjective pain and improved sleep, palpation still indicated the presence of hypervigilance.

**Figure 6:**
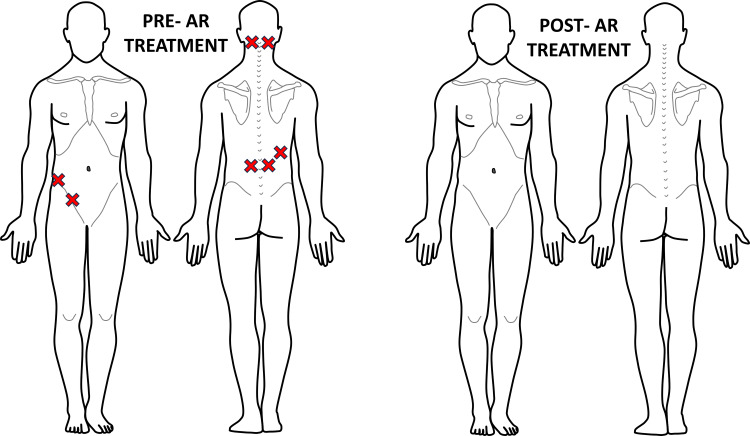
Recording of Case #2 areas of palpatory pain pre- and post-second AR treatment This patient returned with seven areas of palpatory pain found pre-AR treatment one day after the initial visit. Post-AR treatment, this patient once again displayed 0 areas of hypervigilance. The figure was conceptualized and created by authors BS and MKP, respectively. AR: Autonomic recalibration

Further AR treatment was administered, resulting in the resolution of all tenderness to palpation, immediate restoration of trunk range of motion within normal limits, and symptom-free in all planes. The patient’s PQ following his second visit was 0/10, and he was instructed to return to full activities as tolerated (Figure 6).

During the third visit, 24 hours later, the patient reported one episode of brief abdominal pain that resolved without medication. PQ upon arrival was 0/10 and the patient reported no morphine had been taken in the previous 48 hours. Objective reassessment demonstrated restored cervical and lumbar range of motion within normal limits and pain-free. NSE showed a reduction in areas of hypervigilance, with tenderness remaining only in the right psoas and right diaphragm. The treatment successfully resolved these areas. Treatment was concluded and the patient was advised to follow up if symptoms recurred.

Case #3

A 56-year-old male presented with a fourteen-year history of chronic right low back pain. The patient had undergone lumbar fusion surgery (L3-4-5) following a work-related hyperextension injury, but the procedure failed to alleviate his symptoms. Subsequent physical therapy, chiropractic care, and multiple epidural injections provided no relief. He was considered disabled and unable to work. The patient reported constant throbbing and intermittent sharp pain in the right lumbar region, accompanied by episodes of right lower extremity weakness and falls that persisted despite previous treatment by a neurologist. He described sleep disturbances, reliance on oxycodone/acetaminophen for five years, and disability further impaired his quality of life. The patient had a history of various physical and emotional traumatic experiences, such as gallbladder removal in adolescence and fractures of the skull and hand.

**Figure 7:**
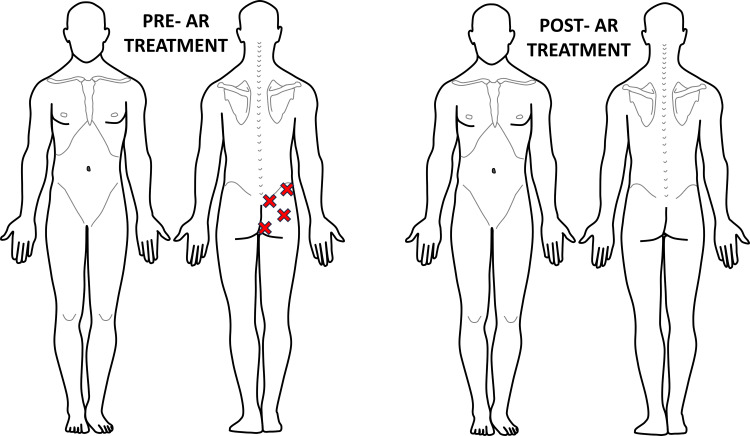
Recording of Case #3 areas of palpatory pain pre- and post-first AR treatment Pre-AR treatment revealed four areas of palpable pain, all of which resolved post-AR treatment. The figure was conceptualized and created by authors BS and MKP, respectively. AR: Autonomic recalibration

Objective evaluations on the first day revealed reduced weight shift to the right during the gait stance phase, limited range of motion and pain in the right hip, knee, and ankle, and significant restrictions and pain during lumbar movements in all planes. Ankle plantarflexion strength was weak, and functional squatting was limited due to pain. NSE identified areas of hypervigilance, particularly in the right coccyx, piriformis, sacroiliac joint, and gluteus maximus. AR treatment to address sympathetic dominance with the PRRT was administered. Residual nociceptive points found at reassessment were resolved with the PNT (Figure 7).

**Figure 8:**
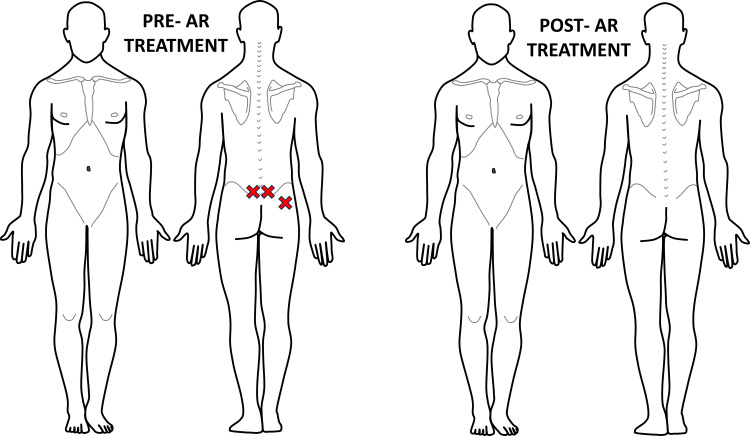
Recording of Case #3 areas of palpatory pain pre- and post-second AR treatment The patient displayed three areas of palpable pain following successful treatment the previous day. Once again, all areas were resolved of palpable pain post-AR treatment. The figure was conceptualized and created by authors BS and MKP, respectively. AR: Autonomic recalibration

Following the first visit, all areas of hypervigilance and pain were resolved. Range of motion in the right hip, knee, and ankle was restored to normal and became pain-free. Lumbar mobility significantly improved, and the patient's wife expressed hope for his ability to return to work. On the second visit, 24 hours later, the patient reported discontinuing oxycodone/acetaminophen and experiencing improved sleep. The patient did not report any withdrawal symptoms to the treating therapist, but it is possible they followed up with their prescribing physician. Objective findings confirmed improved gait, restored joint range of motion, and reduced pain (Figure 8). Additional treatment on the second day addressed remaining restrictions and tenderness, resulting in further pain relief and improved functionality.

During a third visit, two weeks later, the patient reported continued improvement in sleep quality and the ability to sleep in his own bed. Although some residual low back pain returned after the second visit, the patient rated it at a 4/10 primarily in the right sacroiliac area. Objective evaluation showed improved trunk range of motion, ankle strength, and functional squatting. Treatment on the third visit provided further pain relief, as indicated by a PQ of 0/10. The patient returned for a fourth visit, one week later, reporting a pain-free period of seven days without the need for pain medication. Some discomfort during prolonged sitting in the car was noted, indicating a potential persistent dural restriction. Evaluations showed improved tenderness and pain-free movements of the lumbar spine and hip. Functional squatting improved significantly, and all areas of palpable pain resolved. The patient reported long-lasting pain relief and discontinued all medications. No withdrawal symptoms were mentioned by the patient, and it is unknown if he followed up with his prescribing physician after treatment.

## Discussion

This case series introduces AR as a novel approach for alleviating chronic myofascial pain, demonstrating its efficacy in diverse cases including pediatric spondylolisthesis, elderly abdominal surgery history, and chronic post-lumbar fusion pain. AR consistently led to substantial pain reduction, improved sleep, and functional restoration, suggesting its promise as an alternative myofascial pain management method.

The ANS significantly influences pain modulation, and ANS dysregulation is associated with chronic pain. Acute pain activates the SNS for analgesia, but prolonged SNS activity or imbalance between the SNS and PNS can induce chronic pain states [2,3]. Likewise, reduced PNS activity correlates with increased chronic pain [4]. Patients in this case series displayed autonomic hypervigilance within myofascial tissues that was effectively treated by AR, aligning with previous studies demonstrating manual interventions' impact on ANS and pain modulation [10-12].

AR combines manual techniques and patient education to address autonomic dysregulation linked to myofascial pain. By identifying sympathetic dominance and using techniques like the PRRT and PNT, AR resets reflex loops and eliminates hypervigilant regions. AR also addresses the role of emotional trauma in autonomic dysregulation, employing patient education and behavioral treatment [19]. AR's theoretical foundation lies in mitigating myofascial pain by resolving neurologically retained threats and promoting ANS homeostasis, potentially maintaining pain relief through neuroplastic adaptations particularly when emotional pain drivers are recognized.

The case reports highlight AR's clinical potential. Case #1 illustrates complete relief and sports resumption in a pediatric patient with spondylolisthesis. Case #2 demonstrates morphine discontinuation and improved sleep in an elderly patient with a history of abdominal surgeries. Case #3 showcases long-lasting pain relief and medication cessation in a patient with chronic post-lumbar fusion pain. AR displays versatility in addressing diverse sources of chronic pain. Additionally, the rapid response, often within a single session, underscores AR's noteworthy potential and warrants further investigation.

Despite promising case reports, limitations must be considered. Future research should delve into AR's mechanisms, including its impact on neuroplasticity and emotional trauma resolution. While the cited literature supports the hypothesis that autonomic dysfunction may be the source of chronic pain in these patients, physiological evidence is needed to substantiate this claim and should be observed in subsequent studies. Other factors, such as diet and exercise habits, should likewise be considered. Long-term sustainability of AR-induced pain relief also needs investigation. Larger-scale studies with control groups are likewise essential to validate AR's efficacy and compare it to existing treatments. In summary, generalizability to a broader patient population requires additional exploration.

## Conclusions

In conclusion, AR presents a promising approach for alleviating chronic pain by addressing autonomic dysregulation. The case series demonstrates its potential effectiveness and emphasizes the need for further research to validate outcomes and explore mechanisms. AR may prove valuable for healthcare providers seeking alternative methods to manage myofascial pain.

## References

[1] Schlereth T, Birklein F (2008). The sympathetic nervous system and pain. Neuromolecular Med.

[2] Woda A, Picard P, Dutheil F (2016). Dysfunctional stress responses in chronic pain. Psychoneuroendocrinology.

[3] Hallman DM, Lyskov E (2012). Autonomic regulation in musculoskeletal pain. Pain in Perspective.

[4] Tracy LM, Ioannou L, Baker KS, Gibson SJ, Georgiou-Karistianis N, Giummarra MJ (2016). Meta-analytic evidence for decreased heart rate variability in chronic pain implicating parasympathetic nervous system dysregulation. Pain.

[5] Hallman DM, Ekman AH, Lyskov E (2014). Changes in physical activity and heart rate variability in chronic neck-shoulder pain: monitoring during work and leisure time. Int Arch Occup Environ Health.

[6] Gandhi RA, Marques JL, Selvarajah D, Emery CJ, Tesfaye S (2010). Painful diabetic neuropathy is associated with greater autonomic dysfunction than painless diabetic neuropathy. Diabetes Care.

[7] Kim J, Loggia ML, Cahalan CM (2015). The somatosensory link in fibromyalgia: functional connectivity of the primary somatosensory cortex is altered by sustained pain and is associated with clinical/autonomic dysfunction. Arthritis Rheumatol.

[8] Cauzzo S, Singh K, Stauder M (2022). Functional connectome of brainstem nuclei involved in autonomic, limbic, pain and sensory processing in living humans from 7 Tesla resting state fMRI. Neuroimage.

[9] Ge HY, Fernández-de-las-Peñas C, Arendt-Nielsen L (2006). Sympathetic facilitation of hyperalgesia evoked from myofascial tender and trigger points in patients with unilateral shoulder pain. Clin Neurophysiol.

[10] Morikawa Y, Takamoto K, Nishimaru H (2017). Compression at myofascial trigger point on chronic neck pain provides pain relief through the prefrontal cortex and autonomic nervous system: a pilot study. Front Neurosci.

[11] Ruffini N, D'Alessandro G, Mariani N, Pollastrelli A, Cardinali L, Cerritelli F (2015). Variations of high frequency parameter of heart rate variability following osteopathic manipulative treatment in healthy subjects compared to control group and sham therapy: randomized controlled trial. Front Neurosci.

[12] Abuín-Porras V, Clemente-Suárez VJ, Jaén-Crespo G, Navarro-Flores E, Pareja-Galeano H, Romero-Morales C (2021). Effect of physiotherapy treatment in the autonomic activation and pain perception in male patients with non-specific subacute low back pain. J Clin Med.

[13] Kalichman L, Ben David C (2017). Effect of self-myofascial release on myofascial pain, muscle flexibility, and strength: a narrative review. J Bodyw Mov Ther.

[14] Gibbons CH (2019). Basics of autonomic nervous system function. Handb Clin Neurol.

[15] Hansberger BL, Baker RT, May J, Nasypany A (2015). A novel approach to treating plantar fasciitis - effects of primal reflex release technique: a case series. Int J Sports Phys Ther.

[16] Albertin ES, Walters M, May J, Baker RT, Nasypany A, Cheatham S (2020). An exploratory case series analysis of the use of Primal Reflex Release Technique™ to improve signs and symptoms of hamstring strain. Int J Sports Phys Ther.

[17] Kaufman S, Cornu-Labat G (2015). PNT pain neutralization technique: an unprecedented revolution in pain management. https://books.google.com/books/about/PNT_Pain_Neutralization_Technique.html?id=q6YCswEACAAJ.

[18] Hobson JA, Pace-Schott EF (2002). The cognitive neuroscience of sleep: neuronal systems, consciousness and learning. Nat Rev Neurosci.

[19] Hänsel A, von Känel R (2008). The ventro-medial prefrontal cortex: a major link between the autonomic nervous system, regulation of emotion, and stress reactivity?. Biopsychosoc Med.

[20] Van Der Kolk BA (2014). The Body Keeps the Score: Brain, Mind, and Body in the Healing of Trauma. https://www.besselvanderkolk.com/resources/the-body-keeps-the-score.

